# Determination of Dodecanol and Short-Chained Ethoxylated Dodecanols by LC–MS/MS (with Electrospray Ionization) After Their Derivatization (with Phenyl Isocyanate)

**DOI:** 10.1007/s11743-017-2015-z

**Published:** 2017-09-09

**Authors:** Joanna Zembrzuska

**Affiliations:** Institute of Chemistry and Technical Electrochemistry, Poznan University of Technology, Berdychowo 4, 60-965 Poznan, Poland

**Keywords:** Alcohol ethoxylates, LC–MS/MS, Derivatization, LLE, River water, Phenyl isocyanate, Dodecanol

## Abstract

**Abstract:**

This report describes the application of LC–MS/MS for the separation of dodecanol (C_12_OH) and homogenous fatty alcohols ethoxylated (AE) containing a dodecyl moiety and 1–9 ethoxy groups. These ethoxylates and free alcohol were derivatized for LC–MS/MS analysis with phenyl isocyanate (PIC). The derivatives of analytes with PIC were separated using a C18 column. Gradient elution with a mixture of ethyl acetate and acetonitrile (5 mM) was employed. The described determination method is characterized by low detection limits (range from 0.005 µg L^−1^ for: C_12_OH, C_12_EO_2–7_ to 1 µg L^−1^ for C_12_EO_1_) and quantification limits (range from 0.01 µg L^−1^ for: C_12_EO_5–7_ to 2 µg L^−1^ for C_12_EO_1_). The developed and validated method was used in combination with liquid–liquid extraction (using ethyl acetate) in order to identify and quantitatively determine the C_12_OH and C_12_EO_1–9_ present in environmental samples collected from Warta river water in Poznan.

**Graphical abstract:**

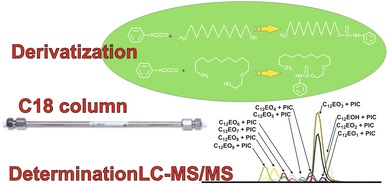

**Electronic supplementary material:**

The online version of this article (doi:10.1007/s11743-017-2015-z) contains supplementary material, which is available to authorized users.

## Introduction

Non-ionic ethoxylated surfactants are common pollutants of the aquatic environment due to their multiple uses as components in the manufacture of laundry detergents, industrial cleaning products and polyurethanes, solubilizers in enhanced oil recovery, emulsifiers in pharmaceutical preparations, additives in cosmetic creams and lotions as well as dispersing and wetting agents [1].

Aliphatic alcohol ethoxylates (AE) are the most important group of non-ionic surfactants and are commonly found in surface water and wastewater [2]. This is associated with the scale of their production, which reached 1 billion tons in 2012 [3]. These compounds are characterized by rapid degradation and low toxicity of some of their biodegradation products, including the formation of free alcohol, ethoxylated polyols and short chain ethoxylated alcohols.

It has been established that AE are degraded according to two dominant mechanisms. The first, which is predominant, is central fission, which results in the formation of a hydrophobic part (free fatty alcohol) and hydrophilic polyethylene glycols (PEGs) [4, 5]. The second mechanism is the stepwise shortening of the ethoxyl chain by removal of single ethoxyl groups [5], mechanism occurring in the environment is associated with Ω-oxidation of the alkyl and ethoxyl part [5, 6]. As a result, ethoxylated alcohols with different ethoxyl chain length, free alcohols and polyethylene glycols are present in wastewater as well as surface water [5, 7].

Analytical methods for the determination of AE include gas chromatography [8], normal-phase high-performance liquid chromatography and reversed-phase HPLC with isocratic and gradient elution [9–11]. Separation of ethoxylated surfactants has been performed using capillary gel electrophoresis (CGE), capillary zone electrophoresis (CZE) [12, 13], micellar electrokinetic chromatography (MEKC) [1], non-aqueous capillary electrophoresis (NACE) [14] and capillary electrochromatography (CEC) [1].

Different detectors can be used for determination of ethoxylated surfactants. The most popular are the ultraviolet detector (UV) [15–17], fluorescence detector [10, 18], flame ionization detector (FID) [19], evaporative light-scattering detector (ELSD) [20, 21], corona-charged aerosol detector (CAD) [1], MS detector and MS/MS detector [6, 11, 18, 22–26].

The lack of a chromophore in the AE molecule in many cases requires the use of a suitable derivatization processes to improve the sensitivity when UV and fluorescence are used. In this case, the most commonly used derivatization agents include 3,5-dinitrobenzoyl chloride [15], naphthyl isocyanate [10, 15], diphenic anhydride [27], naphthoyl chloride [10], phenyl isocyanate [16, 17, 28].

The use of a MS or MS/MS detector for quantitative or qualitative determination of AE, which contain two or more ethoxy groups in their structure, does not require the derivatization process [23]. Unfortunately, due to their structure, the free alcohol and AE with a single ethoxy group (1 or 2) are unstable in the ESI ion source and therefore it is not possible to observe them in the mass spectrum. Therefore, studies regarding the determination of AE after biodegradation processes focused only on the percentage content of these compounds [7]. Most publications are focused on the evaluation of AE exposure in environmental compartments. In almost all of these methods, free alcohols (AO) and low ethoxymers are not detected.

As a result, the above-mentioned compounds should be subjected to a derivatization process prior to their introduction into the LC–MS or LC–MS/MS system, in order to allow for the determination of their presence and subsequent analysis of their quantitative changes during the biodegradation process. Such an approach allows one to determine the content of specific AE including a 12 carbon atom hydrophobic chain connected with a hydrophile which included from 0 to 9 ethoxyl groups.

Over the years, chemical derivatization techniques were developed to enable the ionization of various analytes in LC–MS [27]. Numerous methods employing LC–MS have been developed for AE analysis without derivatization [6, 24, 25, 29, 30] and after derivatization [7, 29, 31, 32].

There are several derivatization agents described in the literature which may be employed for the determination of ethoxylates with ESI-LC–MS. Dunphy *et al*. [33] used 2-fluoro-*N*-methylpyridinium salts as derivatizating agents for determination of free alcohols and low ethoxymers. The group of Ramis-Ramos [34] has published interesting applications for the following analytical characterization reagents: maleic anhydride, phthalic anhydride, and diphenic anhydride. Sparham *et al*. [35] and Cassani *et al*. [36] determined the total amount of AE in the form of 1-naphthoyl chloride derivatives using the LC–MS/MS technique. A quantitative determination of dodecanol and C_12_EO_1–6_ using the LC–MS/MS technique in MRM mode was described by Zgola-Grzeskowiak *et al*. [18]. In this case, 1-naphthoyl chloride was also used as the derivatization agent.

Phenyl isocyanate (PIC) was proposed previously as a derivatizating agent. PIC was used to derivatize AE in raw and treated sewage and river water [16] and was also often used to derivatize poly(ethylene glycol)s [1, 3]. Detection of AE derivatives with phenyl isocyanate was conducted using an UV detector at 240 nm [16, 33]. There are currently no reports regarding detection with the use of MS/MS techniques in the MRM mode. This technique allows for sensitive and selective determination of free alcohol and single ethoxylated alcohols, due to the use of MRM mode.

The use of derivatization during LC–MS/MS determination allows to increase the mass of the pseudomolecular ion, improves its stability in the ion source and enhances the ionization efficiency. As a result, it is possible to determine weakly ionized analytes.

In studies in which PIC was used during the derivatization of AE for their chromatographic determination with UV detection in wastewater, it was observed that signals of derivatives of other contaminants, which were also present in the sample, are visible on the chromatogram. The signals often prevented or hampered the separation of AE [16]. This problem is eliminated by employing the LC–MS/MS method working in the MRM mode.

The aim of this study was to explore the possibility of using PIC as a derivatization agent for quantitative determination of free alcohol and AE with 1–9 ethoxy groups using the LC–MS/MS technique.

The selection of the C_12_EO_*x*_ series is not random, since this series is formed both from renewable and petrochemical sources. Therefore, it is an adequate fingerprint which describes the level of water contamination with non-ionic surfactants [37]. Another reason to direct the focus on the C_12_EO_1–9_ series and dodecanol is the availability of homologous reference standards of this series. Unfortunately, the reference standards for homologues with a higher amount of ethoxyl groups in the compound are not available to date.

## Experimental

### Reagents and Chemicals

Individual alcohol ethoxylates C_12_EO_*X*_ (*x* = 1; 2; 3; 4; 5; 6; 7; 8; 9) and C_12_OH used as standards were obtained from Fluka (Germany), and C_12_EO_3_ was from Sigma-Aldrich (ST. Louis, MO, USA). All standards were of high purity grade (>98%). MS-grade acetonitrile and MS-grade methanol were from Sigma-Aldrich (ST. Louis, MO, USA). Ammonium acetate used as mobile phase additive was purchased from Sigma-Aldrich (ST. Louis, MO, USA). Water was prepared by reverse osmosis in a Demiwa system via double distillation in a quartz apparatus. Only freshly distilled water was used. Phenyl isocyanate (>99%) used as a derivatization agent was purchased from Fluka (Germany).

Ethyl acetate, sodium chloride and hydrogen carbonate (purchased from POCh Gliwice Poland) used for liquid–liquid extraction (LLE) were of analytical grade.

Standard stock solutions (of each compound) were prepared in acetonitrile and kept at 4 °C.

### Derivatization

**Fig. 1:**
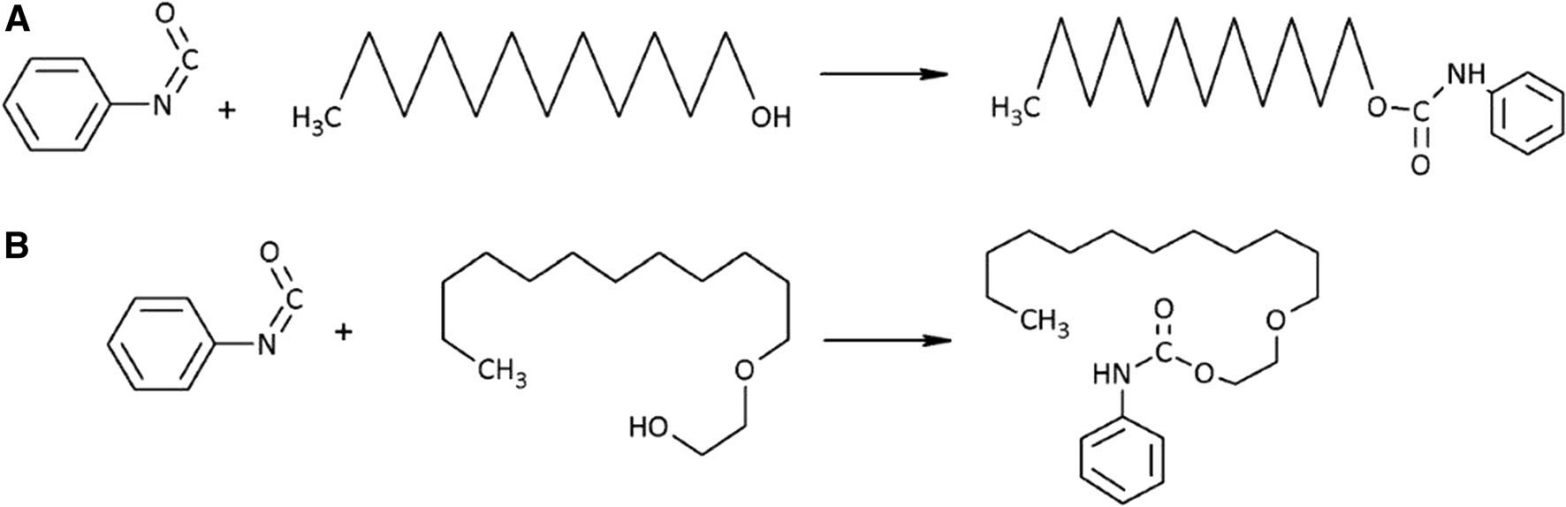
Derivatization reactions of C_12_OH (**a**) and C_12_EO_1_ (**b**) with phenyl isocyanate

PIC (25.5 µL) was added to a sample containing 10 µg of C_12_OH and C_12_EO_1–9_ in 200 µL of acetonitrile. The solution was heated at 70 °C for 30 min. Then, 10 µL of methanol was added and the sample was heated again at 70 °C for 30 min. Finally, the solution was evaporated by increasing the temperature to 80 °C. The dry residue was re-suspended in the mobile phase to a final volume of 1 mL and filtered through a syringe PTFE filter before subjecting it to LC–MS/MS. Examples of the derivatization reactions of C_12_OH and C_12_EO_1_ with phenyl isocyanate are shown in Fig. 1a, b, respectively.

#### Optimization of the Derivatization Procedure

The following parameters were studied during the optimization of the derivatization procedure: time of the derivatization process (from 10 to 60 min, with a 10-min interval), temperature (from 30 to 90 °C with a 10 °C interval) and the amount of the derivatization agent relative to AO and AE (1:1 with a 25, 50 and 100% excess). Each of the samples with optimized parameters was prepared in three replicates.

#### Liquid–Liquid Extraction (LLE)

The procedure of liquid–liquid extraction of AE was previously described by Zembrzuska *et al*. [23]. 15 g of sodium chloride and 0.1 g of sodium hydrogen carbonate were added to 50 mL of the water sample. The sample was extracted with three portions (10, 10 and 5 mL respectively) of ethyl acetate. An aliquot (5 mL) of the combined extracts was evaporated under a gentle stream of nitrogen, reconstituted in acetonitrile and derivatized. This procedure was employed in order to separate and determine the C_12_OH and C_12_EO_1–9_ in water samples collected from the Warta river. The water samples were collected from the Warta river (in Poznan at the measurement and control point on St. Roch Bridge) in accordance with the Polish standard PN EN ISO 566-6:2003. The collecting point was situated upstream of the effluent of the Kozieglowy sewage treatment plant. Samples were collected from the main stream of the river from a depth of 1.0 m. Immediately after transportation to the laboratory in the clean amber glass bottles, the samples were filtered to remove suspended solids using quantitative paper filters, and then subjected to liquid–liquid extraction using ethyl acetate as the extracting agent. The bottles and paper filters were seasoned by an addition of a portion of water in order to prevent adsorptive loss. The extraction was performed immediately after collection of samples (the sampling point was near the laboratory).

Samples were collected in March 2015. Fifty milliliters of each water sample was extracted. The extraction of water and water spiked standard mixture at three different concentrations (2, 4 and 8 µg L^−1^) was carried out. The samples used for the procedure of extraction and analysis of river water samples were prepared in 3 replicates.

### Liquid Chromatography-Tandem Mass Spectrometry

LC analysis was performed using the UltiMate 3000 RSLC chromatographic system from Dionex (Sunnyvale, CA, USA). Five µL samples were injected into an analytical column 100 mm × 2.1 mm I.D packed with 1.9 µm Hypersil Gold C18 RP from Thermo Scientific (USA). The column was heated to 35 °C. The mobile phase was composed of 5 mM ammonium acetate in water (A) and acetonitrile (B), at a flow rate of 0.2 mL/min. The gradient elution was: 0 min 40% B, 10 min 60% B, 20 min 100% B, 25 min 100% B. The LC system was connected to the API 4000 QTRAP triple quadrupole mass spectrometer produced by AB Sciex (Foster City, CA, USA). The Turbo Ion Spray source operated in positive ion mode. Dodecanol and all alcohol ethoxylates were detected using multiple reaction monitoring mode (MRM). ESI ion source parameters were: curtain gas 10 psi, nebulizer gas 40 psi, auxiliary gas 45 psi, temperature 350 °C, ion spray voltage 5500 V and collision gas set to medium. Table 1 shows the MS/MS parameters used for quantitative dodecanol and AE determination. In the optimized conditions, the [M+H]^+^ adducts of C_12_OH, C_12_EO_1–3_ and C_12_EO_8–9_, and [M+NH_4_]^+^ adducts of C_12_EO_4–7_ were used as precursor ions.

**Table 1:** MS/MS parameters for the acquisition of dodecanol and alcohol ethoxylates

Compound	Precursor ion [M+H]^+^ *m/z*	Declustering potential [V]	MRM 1^a^	Collision energy [V]	MRM 2^b^	Collision energy [V]
C_12_OH^c^	306	76	306 → 138	17	–	–
C_12_EO_1_^c^	350	90	350 → 164	19	–	–
C_12_EO_2_^c^	394	106	394 → 164	23	394 → 275	15
C_12_EO_3_^c^	438	96	438 → 319	17	438 → 164	25
C_12_EO_4_	482	41	482 → 271	13	482 → 120	41
C_12_EO_5_^c^	526	71	526 → 407	19	526 → 164	35
C_12_EO_6_	570	86	570 → 451	19	570 → 164	27
C_12_EO_7_	614	136	614 → 495	21	614 → 460	23
C_12_EO_8_^c^	658	121	658 → 540	21	658 → 164	29
C_12_EO_9_^c^	702	126	702 → 584	27	702 → 164	27

Compound	Precursor ion [M+NH_4_]^+^ m/z	Declustering potential [V]	MRM 1^a^	Collision energy [V]	MRM 2^b^	Collision energy [V]
C_12_EO_2_	411	48	411 → 395	11	411 → 275	20
C_12_EO_3_	455	56	455 → 320	19	455 → 439	14
C_12_EO_4_^c^	499	50	499 → 364	23	499 → 164	23
C_12_EO_5_	543	110	543 → 527	20	543 → 408	24
C_12_EO_6_^c^	587	70	587 → 452	26	587 → 571	21
C_12_EO_7_^c^	631	96	631 → 496	28	631 → 164	22
C_12_EO_8_	675	99	675 → 541	28	675 → 164	35
C_12_EO_9_	719	90	719 → 584	29	719 → 164	37

^a^ Detection and quantification transitions

^b^ Confirmation transitions

^c^ In the majority of experiments, complexes of AOH and AE were used as precursor ions

#### Method Validation

The limits of detection (LOD) for each derivative of AE homologues and AO, defined as the concentrations which yielded S/N ratios greater than or equal to 3, and the limits of quantification (LOQ), defined as the concentrations which yielded S/N ratios greater than or equal to 10, were determined for AO and AE homologues in the mobile phase (the solvent used for the introduction of dodecanol and ethoxylates into the LC–MS/MS system) [38]. In order to determine LOD and LOQ, the peak height above the baseline was measured (signal). The peak-to-peak (minimum to maximum) value of baseline noise away from the peak tails for 60 s before the peak was measured [39]. Each analysis was carried out in 3 replicates.

Method limits of detection (MDL) and method limit of quantification (MQL) were calculated based on LOD and LOQ taking into account the concentration factors from river water extraction procedure and the sample dilution factors.

In order to check linearity, mixtures of standards were prepared with the concentration of each component in the range of 0.005–1000 μg L^−1^.

## Results and Discussion

### Optimization of MS/MS Conditions

The first stage of the study aimed to determine the type of ions which could be for detection of C_12_OH and AEO_1–9_ derivatives with PIC. By using ammonium acetate as an addition to the phase, it was possible to obtain [M+NH_4_]^+^ ions as well as [M+H]^+^ ions. In case of C_12_OH and AEO_1_ only the presence of [M+H]^+^ ions was observed in the mass spectrum. Both adducts were visible on the mass spectrum for the remaining AE. Therefore, MRM chromatograms with protonated or ammoniated adducts as precursor ions were obtained initially (Fig. 1S—see Supporting Information). The peaks of adducts which served as pseudomolecular ions in quantitative and qualitative analyzes of actual samples were based on the chromatographic peak areas for specific derivatives of AEO_2–9_. In case of C_12_EO_4_, C_12_EO_6_, C_12_EO_7_ these were [M+NH_4_]^+^ ions, whereas [M+H]^+^ were used for C_12_EO_2_, C_12_EO_3_, C_12_EO_5_, C_12_EO_8_ and C_12_EO_9_.

### Fragmentation of AE Derivatives with PIC

**Fig. 2:**
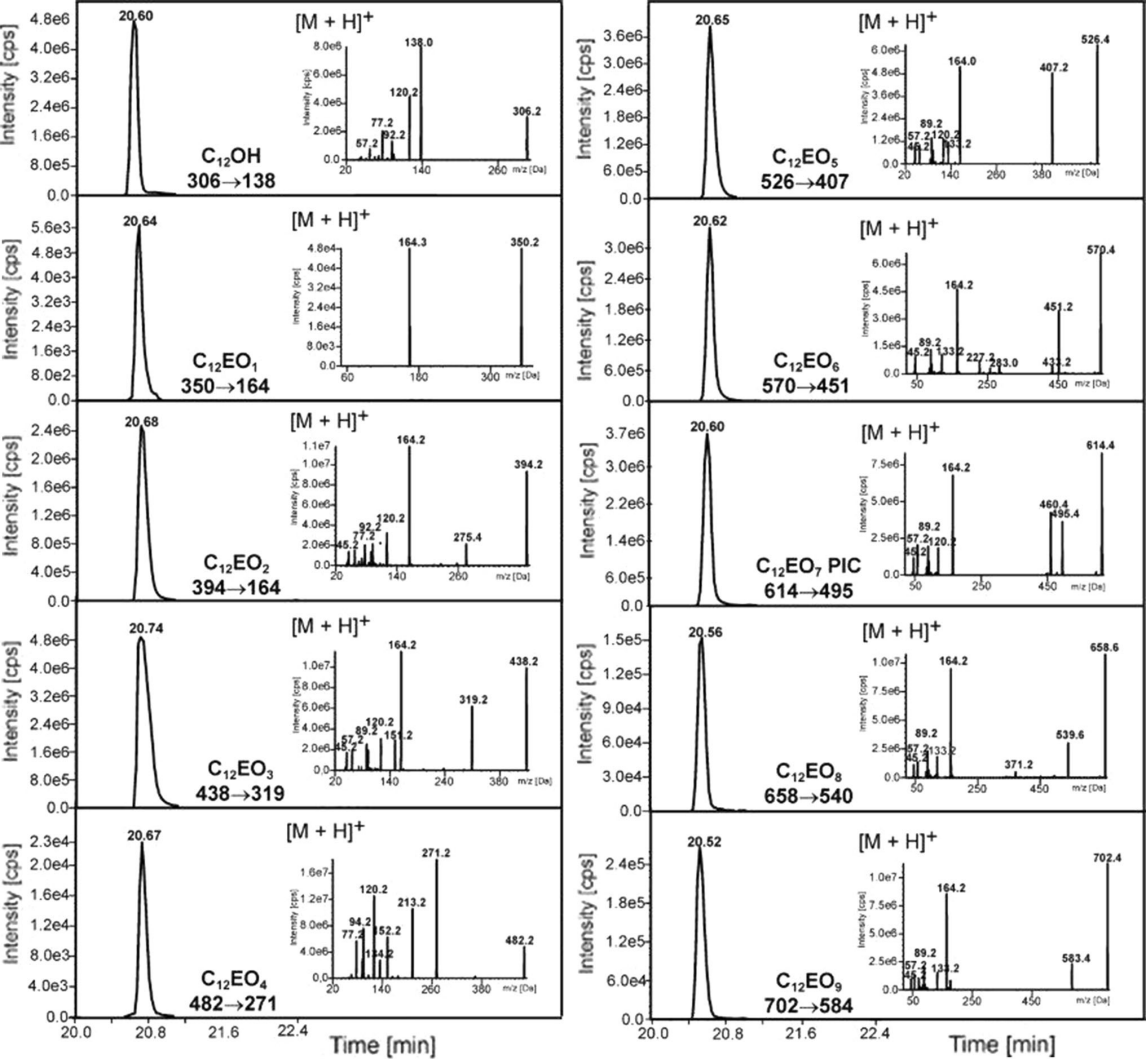
Mass chromatograms of MRM pairs and fragmentation spectra originating from dodecanol and AE with PIC for protonated ions [M+H]^+^ at the concentration of 1 g mL^−1^

**Fig. 3:**
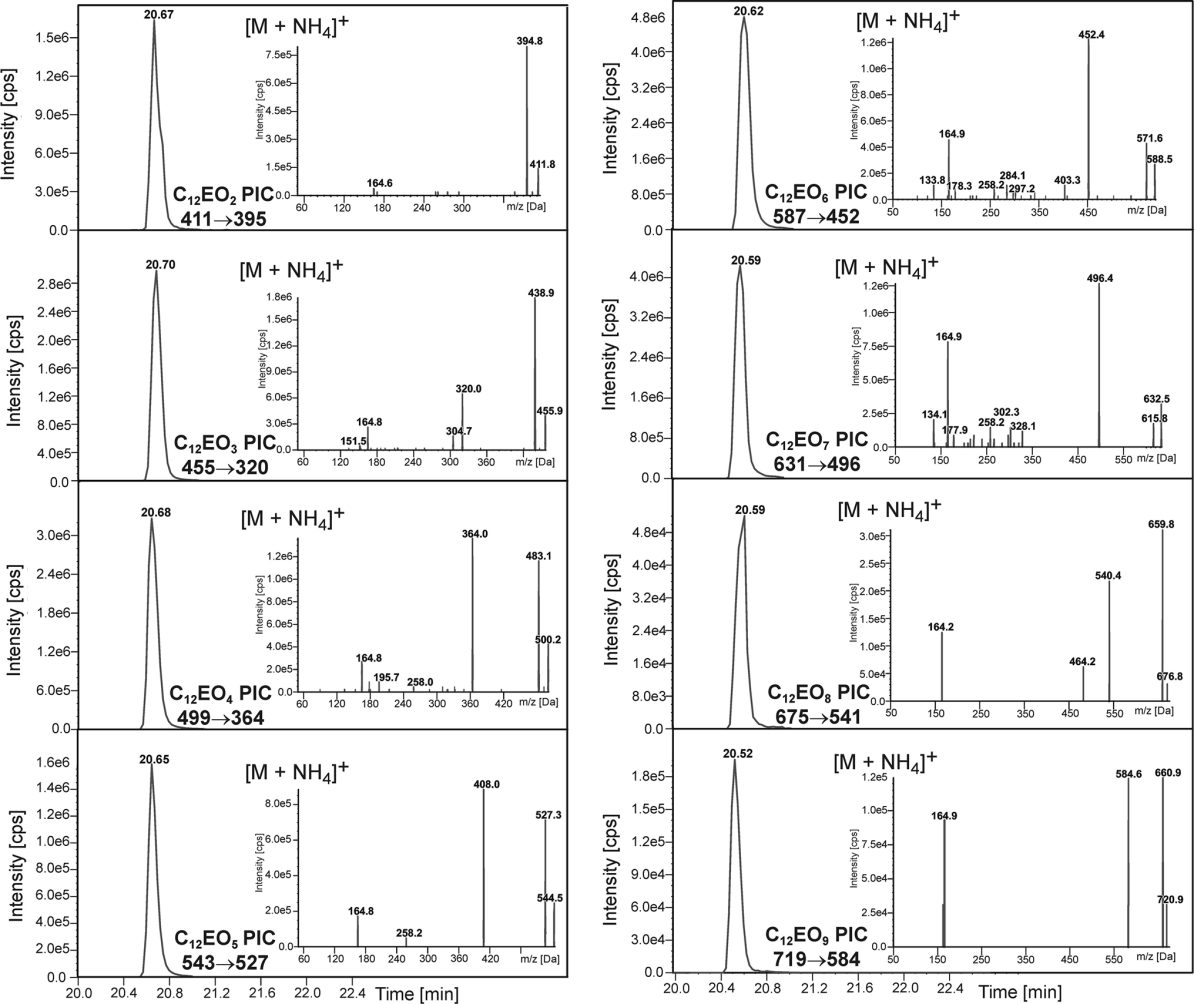
Mass chromatograms of MRM pairs and fragmentation spectra originating from dodecanol and AE with PIC for ammoniated ions [M+NH_4_]^+^ at the concentration of 1 g mL^−1^

Fragmentation of protonated or ammoniated derivatives of C_12_EO_1–9_ homologues and C_12_OH was investigated. Collision conditions were optimized automatically by the spectrometer program and are given in Table 1. The results, together with chromatograms with MRM detection, are shown in Figs. 2 and 3. In case of C_12_OH and C_12_EO_1_ only the detection of protonated adducts was possible and a single transfer between the pseudomolecular ion and the fragmentation ion was determined.

The fragmentation spectra of ammoniated ions (Fig. 3) differ from the spectra of protonated ions due to the presence of one additional fragment, i.e. the formation of a protonated ion. The signals with the highest intensity, which are visible on the fragmentation spectra, originate from the protonated ions of AE derivatives with PIC [M+H]^+^, e.g. C_12_EO_5_ − *m*/*z* = 526 (Figs. 2, 3); whereas the signals with lower intensity occur due to the loss of phenyl isocyanate or subsequent ethoxy groups from the AE molecule. The signal at m/z = 164, which is present on every spectrum, occurs due to the loss of a fragment of the phenyl isocyanate –OCH_2_CH_2_– unit, e.g. in case of C_12_EO_5_ this corresponds to a transfer from *m*/*z* = 526 to *m*/*z* = 164 (loss of a unit with *m*/*z* = 362). The repeatedly appearing signal at *m*/*z* = 77 originates from the cleavage of the benzene ring in the PIC unit, whereas the signal at *m*/*z* = 45 originates from HOCH_2_CH_2_· ethoxyl groups.

### Optimization of the Derivatization Procedure

In order to ensure that the derivatization procedure is repeatable and efficient, it has been optimized in terms of reaction temperature, reaction time and the amount of the derivatization agent. All the studies were conducted for a mixture of C_12_OH and C_12_EO_1–9_.

**Fig. 4:**
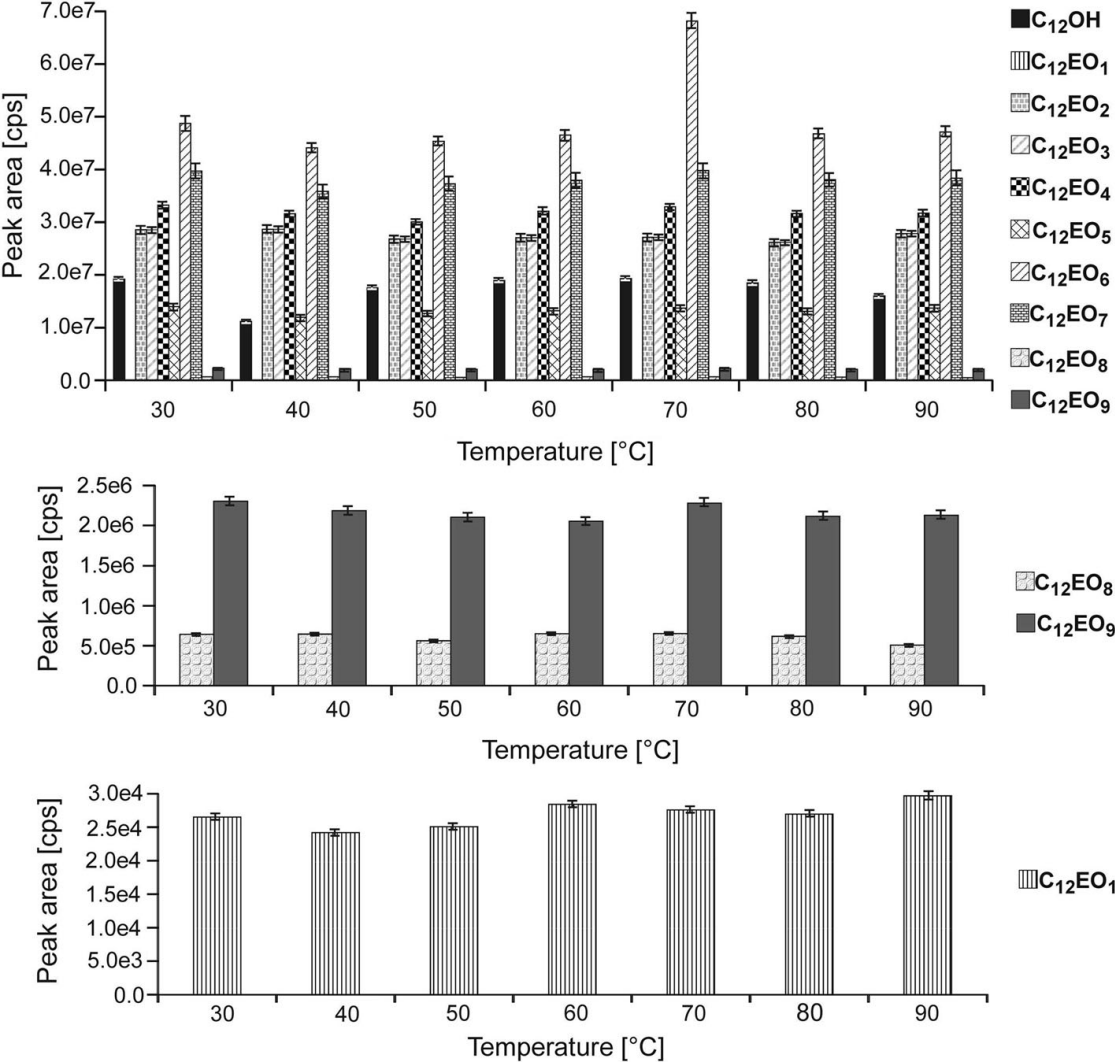
Influence of the temperature of derivatization reaction on the peak areas for derivatives of C_12_OH and C_12_EO_1–9_ with PIC. *Asterisk* the *bars* in the graph are standard *error bars*

During the first step, the influence of temperature on the formation of derivatives was analyzed. The reaction was carried out at temperature values ranging from 30 to 90 °C with a 10 °C interval. It was observed that the derivatization process may be conducted at 30 °C for C_12_OH, C_12_EO_2_, C_12_EO_3_, C_12_EO_4_, C_12_EO_5_, C_12_EO_7_, C_12_EO_8_ and C_12_EO_9_, since the highest peak areas were obtained for these compounds at these temperature values (Fig. 4). For each compound, with the exception of C_12_EO_6_, the increase in temperature did not significantly impact the increase or decrease in the peak area. It was also observed that the temperature of 70 °C is optimal for each of the studied C_12_EO_1–2_ homologues as well as C_12_OH, since the derivatization process at this temperature value resulted in the highest peak areas. This is also the only value which allowed for maximum peak areas in the case of C_12_EO_6_. Therefore, the derivatization reaction was carried out at the temperature of 70 °C during subsequent studies.

**Fig. 5:**
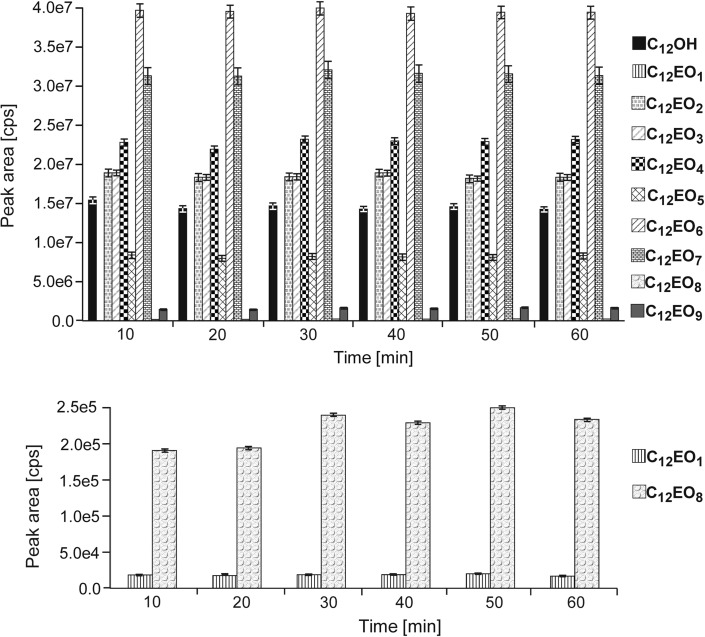
Influence of the time of derivatization reaction on the peak areas for derivatives of C_12_OH and C_12_EO_1–9_ with PIC. *Asterisk* The *bars* in the graph are standard *error bars*

The next optimization step was focused on the influence of time on the derivatization process. The derivatization reaction was conducted for periods of 10, 20, 30, 40, 50 and 60 min. Based on the obtained peak area values for all the studied compounds (which are shown on Fig. 5), it was established that time = 30 min is optimal for the derivatization process. After this time, the maximum values of peak areas were achieved for each component present in the studied mixture. It was also observed that in the case of C_12_OH, C_12_EO_2_, C_12_EO_3_, C_12_EO_4_, C_12_EO_5_, C_12_EO_6_ and C_12_EO_7_ the reaction time did not influence the efficiency of the derivatization process. In the case of the remaining homologues, a reaction time lower than 30 min resulted in decreased peak areas. Based on the results obtained during this step, a derivatization time of 30 min was used during subsequent studies.

**Fig. 6:**
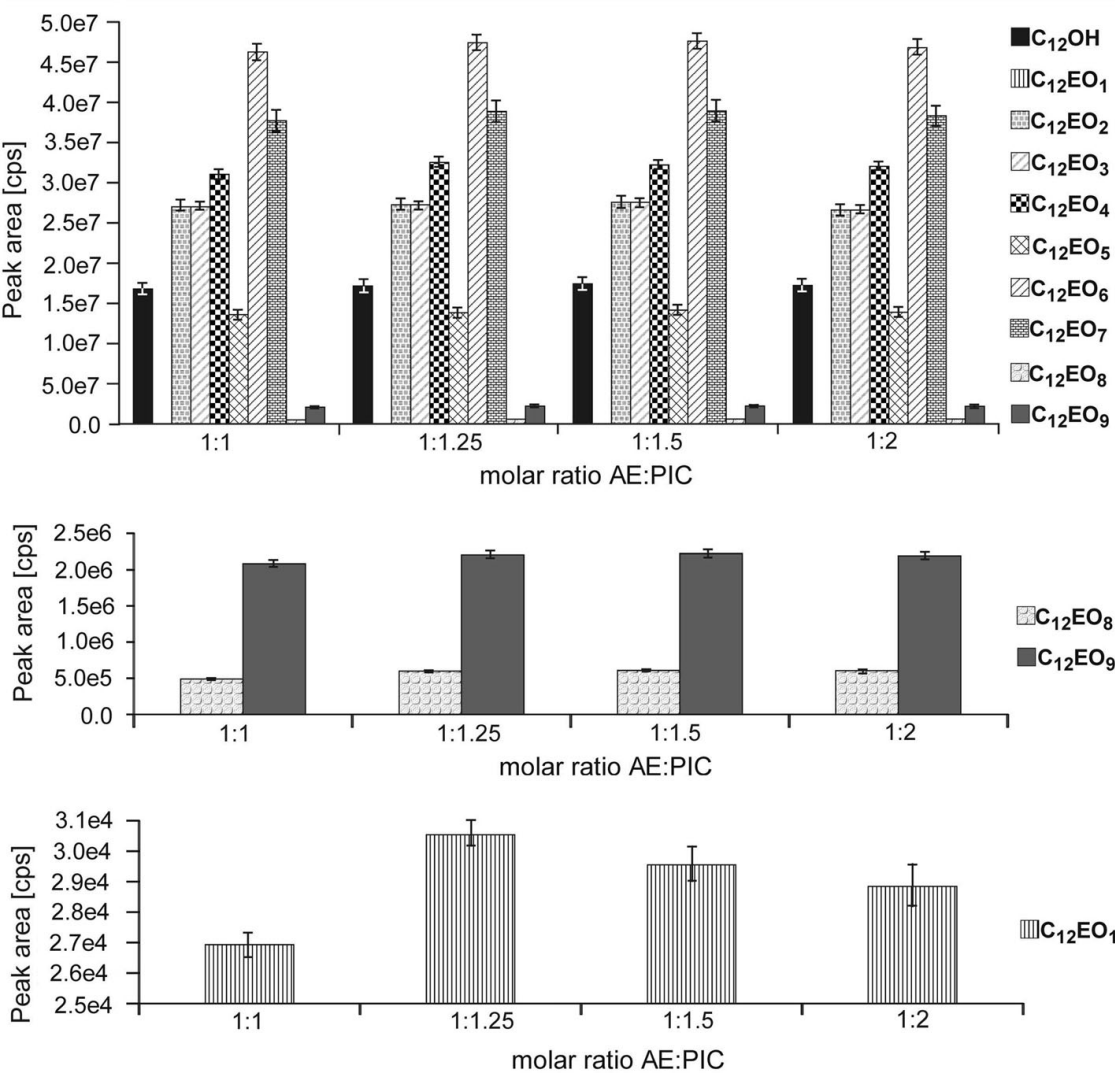
Influence of the amount of derivatization agent of derivatization reaction on the peak areas for derivatives of C_12_OH and C_12_EO_1–9_ with PIC. *Asterisk* The *bars* in the graph are standard error bars

The last optimization step was focused on the influence of the amount of derivatization agent relative to the studied mixture. The experiments were carried out using equimolar ratios of each component of the mixture relative to PIC (1:1) and different molar ratios: 1:1.25 (25% excess of PIC), 1:1.5 (50% excess of PIC) and 1:2 (100% excess of PIC). On the basis of peak areas obtained for all studied compounds (Fig. 6), it was established that a 50% excess of the derivatization agent allowed to achieve maximum peak areas of each compound. Based on the analysis of Fig. 6, it was also observed that in case of C_12_OH, C_12_EO_2_, C_12_EO_3_, C_12_EO_5_, C_12_EO_6_ and C_12_EO_7_ maximum peak areas may be achieved during derivatization without any excess of PIC and that these values do not change when excess of the derivatization agent is used. In case of the remaining compounds an excessive amount of PIC is necessary in order to achieve maximum peak areas. Based on the obtained results, a 50% excess of PIC relative to each mixture component was used during subsequent studies (the reaction was carried out at a 1:1.5 ratio).

### Method Validation

Linearity, LOD and LOQ were determined for [M+H]^+^ ions and [M+NH_4_]^+^ ions (see Table 2). Linearity was tested by analyzing free dodecanol samples and ethoxylated alcohol at different concentrations, ranging from 0.005 to 1000 µg L^−1^. Good linearity was achieved for all compounds, with correlation coefficients >0.97 (see Table 2). In the studied concentration range only C_12_EO_8_ and C_12_EO_9_ exhibited a single linearity range for protonated adducts. The remaining protonated or ammoniated adducts of C_12_OH and AE derivatives exhibited two linearity ranges in the studied concentration range. The LOD ranged from 0.005 µg L^−1^ for C_12_OH and C_12_EO_2–7_ to 1 µg L^−1^ for C_12_EO_1_. The LOQ ranged from 0.01 µg L^−1^ for C_12_EO_5–7_ to 2 µg L^−1^ for C_12_EO_1_. The MQL (method limit of quantification) values for river water samples ranged from 0.0008 µg L^−1^ for C_12_EO_5_ and C_12_EO_6_ to 0.1 µg L^−1^ for C_12_EO_1_, whereas MDL (method limit of detection) values ranged from 0.0007 µg L^−1^ for C_12_EO_8_ to 0.06 µg L^−1^ for C_12_EO_1_. After considering the injection volume, which amounted to 10 µL, the MDL values range from 0.007 pg for C_12_EO_8_ to 0.6 pg for C_12_OH. The MDL values obtained for river water are at least one order of magnitude lower compared to those obtained by Crescenzi *et al*. [30]. It should be noted, that the MDL depends on the matrix as well as separation and concentration techniques which were used during sample preparation. Crescenzi *et al*. [30] separated the ethoxylates using SPE on a GCB column, while the LOD and LOQ were determined against an internal standard (C_10_EO_6_) based on a single chromatographic peak for the complete C_12_EO_*x*_ series obtained in TIC (total ion current) mode. The identification of the mixture was conducted by analysis of mass spectrum for this peak. Therefore, the determined LOD and LOQ values as well as concentrations in the actual samples are for the sum of the total C_12_EO_*x*_ series, not for specific ethoxylated alcohols in the C_12_EO_*x*_ series, as in this study. The presented study employed classic LLE technique with ethyl acetate, and the LOD, LOQ values as well as concentrations of C_12_OH and C_12_EO_1–9_ in river water were determined using the analyzed compounds as standards (the areas of chromatographic peaks). Each of the determined ethoxylated alcohols was a separated, single peak, due to the use of single homologues and the MRM mode during their detection with the MS/MS technique and prior derivatization with the use of PIC. The only limitation of the presented methodology is the lack of single homologues with more than 9 ethoxyl groups in the structure of the C_12_EO_x_ compound series on the market, since such compounds are also present in surface water as confirmed by literature references [2].

**Table 2:** Validation data for determination of C_12_OH and C_12_EO_1–9_

Compound	Calibration curve range (µg L^−1^)	Correlation coefficient (*r* ^2^)	Calibration curve range (µg L^−1^)	Correlation coefficient (*r* ^2^)	LOD (µg L^−1^)	MDL (µg L^−1^)	LOQ (µg L^−1^)	MQL (µg L^−1^)
C_12_OH [M+H]^+^	0.05–200	0.9984	200–1000	0.8707	0.005	0.0004	0.05	0.004
C_12_EO_1_ [M+H]^+^	2–200	0.9997	200–1000	0.9943	1	0.06	2	0.1
C_12_EO_2_ [M+H]^+^	0.05–200	0.9988	200–1000	0.9882	0.005	0.0005	0.05	0.005
C_12_EO_3_ [M+H]^+^	0.02–200	0.9983	200–1000	0.9918	0.005	0.0004	0.02	0.002
C_12_EO_4_ [M+NH_4_]^+^	0.05–200	0.9992	200–1000	0.9900	0.005	0.0004	0.05	0.004
C_12_EO_5_ [M+H]^+^	0.01–200	0.9988	200–1000	0.9691	0.005	0.0004	0.01	0.0008
C_12_EO_6_ [M+NH_4_]^+^	0.01–200	0.9992	200–1000	0.9706	0.005	0.0004	0.01	0.0008
C_12_EO_7_ [M+NH_4_]^+^	0.01–200	0.9992	200–1000	0.9928	0.005	0.0006	0.01	0.001
C_12_EO_8_ [M+H]^+^	0.05–1000	0.9971	–	–	0.01	0.0007	0.05	0.004
C_12_EO_9_ [M+H]^+^	0.01–1000	0.9982	–	–	0.005	0.0004	0.01	0.007

The LOD values are not equal for all the studied ethoxylated alcohol homologues. Such differences result from different responses of the detector, which are influenced by ionization. Such dependencies were observed during direct determination (without derivatization) of these compounds using the LC–MS/MS technique [23]. Perhaps the length of the ethoxyl chain influences the ionization process. With the increase of the ethoxyl chain length up to a certain value more stable complexes are formed. These observations were also described by Crescenzi *et al*. [30]. The authors observed that the response of the detector increased exponentially with the increase of the amount of ethoxyl groups in the compounds from 1 to 6. This dependency was not observed for ethoxylated alcohols with a number of ethoxyl groups higher than 6.

The determined LOD and LOQ values for AE derivatives with phenyl isocyanate were lower by two orders of magnitude compared to values obtained for the direct determination method of these compounds using LC–MS/MS in the form of protonated adducts [23]. Additionally, the LOD values obtained in the framework of this study were lower by one order of magnitude compared to LOD values obtained for derivatives of and C_12_EO_1–6_ with 1-naphthoyl chloride using the same technique [18].

### River Water Sample

In order to check whether the described method may be used for determination of C_12_OH and C_12_EO_1–9_ in actual environmental samples, the compounds were determined in a non-spiked and spiked (at three concentration levels: 2, 4 and 8 µg L^−1^) river water samples (River Warta, Poznan, Poland). Ethyl acetate was used for LLE. Separation and determination was performed in accordance with sections *Derivatization* and *Liquid chromatography*-*tandem mass spectrometry*. The results are shown in Table 3. The concentration values of specific analytes present in the non-spiked Warta river water samples were determined using the multiple standard addition method [37].

**Table 3:** Concentrations of C_12_OH and homogeneous AE in Warta River in March 2015, and recoveries of spikes of C_12_OH and C_12_EO_1–9_ Warta River

Homologue	River water found	Spike 2 µg L^−1^		Spike 4 µg L^−1^		Spike 8µg L^−1^	
	(µg L^−1^) ± SD	Found (µg L^−1^) ± SD	Recovery (%)	Found (µg L^−1^) ± SD	Recovery (%)	Found (µg L^−1^) ± SD	Recovery (%)
C_12_OH	10.00 ± 0.28	10.32 ± 0.28	16.0	12.04 ± 0.33	51.0	16.24 ± 0.35	78.0
C_12_EO_1_	2.19 ± 0.04	2.75 ± 0.07	28.0	3.98 ± 0.07	44.8	8.66 ± 0.15	80.9
C_12_EO_2_	1.20 ± 0.03	2.18 ± 0.06	49.0	4.37 ± 0.11	79.3	8.53 ± 0.23	91.6
C_12_EO_3_	0.56 ± 0.01	1.59 ± 0.03	51.5	3.84 ± 0.07	82.0	7.98 ± 0.11	92.8
C_12_EO_4_	0.55 ± 0.01	1.82 ± 0.03	63.5	3.84 ± 0.07	82.3	7.72 ± 0.11	89.6
C_12_EO_5_	0.36 ± 0.02	1.85 ± 0.08	74.5	3.87 ± 0.18	87.8	7.51 ± 0.34	89.4
C_12_EO_6_	0.06 ± 0.01	1.33 ± 0.03	63.5	3.07 ± 0.07	72.3	6.84 ± 0.13	84.8
C_12_EO_7_	0.06 ± 0.01	1.28 ± 0.04	61.0	3.15 ± 0.11	77.3	6.79 ± 0.22	84.1
C_12_EO_8_	0.24 ± 0.01	1.66 ± 0.01	71.0	3.37 ± 0.02	78.3	6.87 ± 0.06	82.9
C_12_EO_9_	0.13 ± 0.01	1.51 ± 0.03	69.0	3.61 ± 0.08	87.0	6.54 ± 0.15	80.1

All of the studied analytes were identified in the Warta river water samples. The highest concentrations were determined for dodecanol (10 μg L^−1^) whereas the lowest were observed for C_12_EO_6_ and C_12_EO_7_ (0.06 μg L^−1^). The obtained concentrations of C_12_EO_2_–C_12_EO_9_ homologues were at a similar level compared to the results described by Zembrzuska *et al*. regarding the monitoring of Warta river during a period from October 2011 to September 2012 [37]. The authors determined non-derivatized AE using LC–MS/MS. It was observed that the obtained recovery rates for higher additions of the model mixture were higher compared to recovery rates obtained for lower additions of the model mixture. It was also observed that in case of additions of 4 and 8 μg L^−1^, the recovery rates increased along with the increase of the ethoxy chain length in the surfactant molecule. The same dependencies, namely higher concentrations of AE containing single ethoxyl groups in the compound and free alcohol, were observed by other research groups [7, 40], which indicates that central fission is the dominating biodegradation mechanism and the source of free alcohol in surface water and treated wastewater. Since the alcohol ethoxylates C_12–13_ with low EO groups are characterized by high toxicity [7, 41], their monitoring is crucial for proper environmental protection. The observation of their concentration in the environment is associated with the use of sensitive, selective analytical methods which allow one to precisely determine their content. The LC–MS/MS in MRM mode may be a solution for determination of AE derivatives with PIC. Employing this technique allows one to eliminate the additional peaks associated with the presence of derivatives of other contaminants in the river water samples, which were observed during UV detection [16].

Due to the low level of analyte and the complex matrix, the use of derivatization allowed to us increase the sensitivity and range of determined AE (with 1–2 ethoxyl groups and free alcohol) in comparison to detection of non-derivatized compounds [23]. Furthermore, the use of derivatization caused an increase in volatility during ionization in the ESI source and changed the behavior of compounds during their chromatographic separation, which allowed to use the C18 column for separation of AE based on the ethoxyl chain length in a reversed phase system, which is not possible without derivatization, MS/MS detection in MRM mode and the use of expensive chromatographic columns [23].

## Concluding Remarks

The described method for determination of dodecanol and C_12_EO_1–9_ in the form of derivatives with phenyl isocyanate using the LC–MS/MS technique is characterized by lower LOD and LOQ values compared to other determination methods of these compounds, in the form of derivatives with other derivatization agents [18] as well as non-derivatized form using LC–MS/MS [23]. In combination with liquid–liquids extraction using ethyl acetate, this method is an appropriate and sensitive analytical tool for the monitoring of environmental water samples.

## Electronic supplementary material

Below is the link to the electronic supplementary material.

Supplementary material 1 (JPEG 389 kb)
