# Supplementary figures and images for: Determination of Dodecanol and Short-Chained Ethoxylated Dodecanols by LC–MS/MS (with Electrospray Ionization) After Their Derivatization (with Phenyl Isocyanate)
Source: J Surfactants Deterg. 2017 Sep 9;20(6):1421–32. doi: 10.1007/s11743-017-2015-z (PMC5686276; doi:10.1007/s11743-017-2015-z)

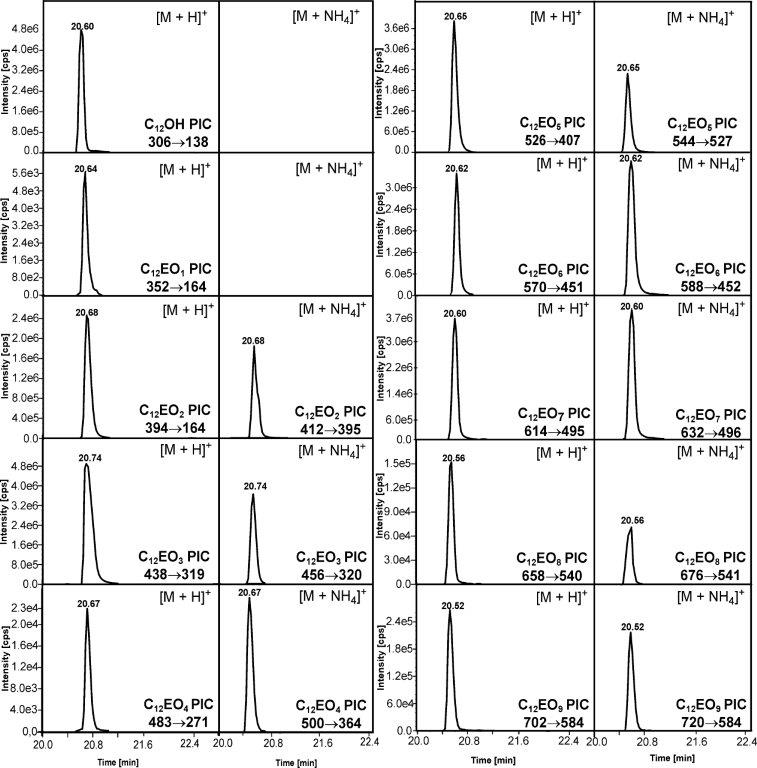

Supplement: Supplementary file 1 — Supplementary material 1 (JPEG 389 kb) [file 11743_2017_2015_MOESM1_ESM.jpg]
